# High-Dose Acetaminophen as a Treatment for Cancer

**DOI:** 10.3390/livers4010007

**Published:** 2024-01-31

**Authors:** Jeffrey Wu, Bradley Maller, Rujul Kaul, Andrea Galabow, Allyn Bryan, Alexander Neuwelt

**Affiliations:** 1 Department of Neurology, School of Medicine, Oregon Health and Sciences University, 3181 Sam Jackson Park Rd., Portland, OR 97239, USA;; 2 Department of Veterans Affairs, 1201 Broad Rock Blvd, Richmond, VA 23249, USA;; 3 Department of Internal Medicine, School of Medicine, Virginia Commonwealth University, 1201 E Marshall St., Richmond, VA 23298, USA

**Keywords:** acetaminophen, N-acetylcysteine, fomepizole, cancer

## Abstract

The use of high-dose acetaminophen (AAP) with n-acetylcysteine (NAC) rescue was studied as an anti-cancer treatment in phase I trials with promising signals of anti-tumor efficacy. Correlative analysis suggested that AAP has a free-radical-independent mechanism of anti-tumor activity—in contrast to the well-established mechanism of AAP hepatotoxicity. Subsequent “reverse translational” studies in the pre-clinical setting have identified novel mechanisms of action of high-dose AAP, including modulation of JAK-STAT signaling in both the tumor cell and the tumor immune microenvironment. Importantly, these effects are free-radical-independent and not reversed by concurrent administration of the established AAP rescue agents fomepizole and NAC. By administering high-dose AAP concurrently with fomepizole and NAC, 100-fold higher AAP levels than those of standard dosing can be achieved in mice without detected toxicity and with substantial anti-tumor efficacy against commonly used mouse models of lung and breast cancer that are resistant to standard first-line anti-cancer therapies. With these recent advances, additional clinical trials of high-dose AAP with concurrent NAC and fomepizole-based rescue are warranted.

## 1. AAP Is Hepatotoxic in Overdose

Acetaminophen (AAP) is a commonly used drug throughout the world, standardly taken as an anti-nociceptive and fever-reducing agent. AAP is metabolized by a variety of pathways, including glucuronidation, sulfation, and fatty acid amide hydrolase (FAAH)-mediated conversion to N-arachidonoylphenolamine (AM404); AM404 is thought to be an active metabolite that contributes to the anti-nociceptive properties of AAP [1]. Additionally, CYP2E1-mediated conversion of AAP into N-acetyl-p-benzoquinone imine (NAPQI) is a minor metabolic pathway, comprising about 5–10% of AAP metabolism [2]. NAPQI is a reactive intermediate that under normal conditions is rapidly bound and detoxified by intracellular glutathione (GSH). However, upon AAP overdose, intracellular GSH becomes depleted and NAPQI binds cellular proteins, predominantly within the liver, resulting in toxicity [3]. Of note, NAPQI bound to cellular proteins, termed protein adducts, can be measured using mass spectrometry and is a relatively specific measure of AAP toxicity [4]. Key evidence for the mechanisms of AAP liver toxicity come from studies demonstrating substantially improved tolerability of high doses of AAP in CYP2E1 KO mice [5].

## 2. Strategies to Prevent AAP Toxicity

The FDA-approved antidote for AAP toxicity is n-acetylcysteine (NAC), which can be administered either orally or intravenously. NAC functions both as a GSH precursor and also as a potent thiol anti-oxidant that binds and detoxifies NAPQI, thus mitigating AAP hepatotoxicity. Clinically, when NAC is administered within 6–8 h of AAP overdose, outcomes are positive the vast majority of the time with a relatively minimal risk of mortality [3,6,7]. Of note, as outlined in a Cochrane database review, the data supporting the use of NAC as a clinical antidote to AAP toxicity are largely based on single-arm, non-randomized studies [8]. Thus, the extent to which patients that overdose on AAP may spontaneously recover is not completely understood.

Fomepizole (4-methylpyrazole, 4-MP) is a potent CYP2E1 inhibitor that is increasingly being utilized as an adjunctive therapy in the management of AAP overdose [9,10]. Fomepizole is FDA-approved for the treatment of ethylene glycol and methanol toxicity and has a relatively well-established safety profile with few significant toxicities [11]. Fomepizole inhibits CYP2E1-mediated NAPQI formation and subsequent free-radical-mediated stress response in the mitochondria upon AAP overdose via inhibition of N-terminal JNK phosphorylation [12]. Fomepizole rescue prevents the formation of oxidative metabolites, such as APAP-Cys, while not affecting other metabolic pathways such as glucuronidation [12].

Numerous other antidotes to AAP toxicity have been evaluated pre-clinically, including 25-hydroxycholesterol-3-sulfate [13] and heparan sulfates [14]. However, these drugs have not been evaluated in human patients for protection against AAP toxicity; fomepizole and NAC are the drugs with the most clinical data supporting their use.

## 3. Early Pre-Clinical Studies of High-Dose AAP for the Treatment of Cancer

The initial pre-clinical studies using high-dose AAP as a treatment for cancer utilized hepatoma cell lines HEPG2 and PLC/PRF/5 [15]. These studies were based on the pre-sumption that n-acetylcysteine functions as an “antagonist” to AAP, i.e., neutralizing both the toxicity and efficacy of AAP. Wu et al. designed experiments with the goal of allowing NAC to selectively rescue normal hepatocytes but not malignant hepatoma cells from high-dose AAP treatment. The experiments took advantage of the presence of receptors for galactose-terminal (asialo-)glycoproteins on normal hepatocytes. A conjugate was created by coupling NAC to galactose-terminal (asialo-)fetuin. It was demonstrated that HEPG2 cells (capable of taking up galactose-terminal (asialo-)fetuin) were effectively rescued by the modified NAC; however, PLC/PRF/5 cells could not take up the galactose-terminal (asialo-)fetuin-bound NAC and thus were not rescued.

## 4. Clinical Trials of High-Dose AAP as a Treatment of Cancer

Clinical trials evaluating high-dose AAP as a treatment for cancer were conceived based on relatively vague principles that AAP may have differential effects on neoplastic cells relative to normal cells, thus permitting selective rescue of normal cells using NAC-based rescue approaches. For instance, the clinical trials cited studies that utilized NAC to blunt the toxicity of alkylating agents and radiation to normal cells without preventing tumoricidal activity [16]. In reality, these clinical trials were designed based on relatively scant pre-clinical rationale and in the absence of in vitro or in vivo mouse studies directly evaluating the anti-tumor properties of high-dose AAP with delayed NAC rescue—the treatment regimen used in the studies [17].

Kobrinsky et al. enrolled 19 patients onto their trial of high-dose AAP from 1990 to 1991 [17]. The patients had a wide range of malignancies, including pancreatic cancer, prostate cancer, and esophageal cancer, among others. The majority of patients had solid tumors, while one patient had acute leukemia. AAP was administered orally as a concentrated slurry. NAC was administered starting 8 h after the AAP treatment using a 16-hour infusion (NAC dosing was similar to what is used in the management of AAP overdose). Delayed NAC rescue was used based on the concern that NAC functions as an “antagonist” of AAP and concurrent administration would therefore neutralize its efficacy. However, no rationale was provided for why delayed NAC would rescue the normal liver but not the cancer.

Treatment with high-dose AAP was performed weekly for 4 weeks and continued until disease progression or unacceptable toxicity. AAP was started at 6 g/m^2^ and escalated up to a maximum of 20 g/m^2^. A total of 78 courses of high-dose AAP were administered. Out of 14 patients assessed for response, 3 had a partial response (responding patients had esophageal cancer, pancreatic cancer, and small-cell lung cancer), 3 were “improved,” and 3 patients had a mixed response. Thus, the overall response rate among assessable patients was 3/14 or 21%. Interestingly, 8 of the patients with chronic pain reported complete analgesic control for 12–24 h after the injection. No dose-limiting liver toxicities were observed. The most common adverse events were nausea (associated with swallowing the large volume of AAP slurry) and drowsiness that usually resolved around the time the NAC infusion was started. In pharmacological analysis, AAP was noted to have a half-life of 3.6 h with mean serum AAP concentration at 4 h of 245 μg/mL (range 95–473 μg/mL).

While the published manuscript was a clinical trial, in the Discussion section it was revealed that “Corden and Bartlett-Heubus have demonstrated a reproducible antiproliferative effect of HDAC on Ll210 leukemia cells in vitro despite no demonstrable decrease in intracellular glutathione [17]”. This observation was the first to suggest a possible free-radical-independent mechanism of anti-tumor activity in high-dose AAP.

A subsequent phase I clinical trial of high-dose AAP was performed by Wolchok et al. in patients with advanced melanoma at Memorial Sloan Kettering Cancer Center [18]. The trial evaluated a combination of AAP and carmustine (BCNU). The authors hypothesized that AAP would deplete tumoral GSH, a molecule known to be involved in chemotherapy resistance, and thus sensitize tumors to BCNU. However, a GSH-mediated mechanism of synergy between AAP and chemotherapy had not yet been tested in animal models.

Wolchok et al. designed the study to sequentially dose-escalate AAP and BCNU. The starting dose of AAP was 10 g/m^2^ every 3 weeks; BCNU was started at 10 mg/m^2^. AAP was escalated by 5 g/m^2^ in cohorts until the DLT (dose-limiting toxicity) was reached. Once the DLT of AAP was reached, BCNU dosing was subsequently elevated using a 3 + 3 design. AAP was again given as a slurry; however, due to nausea and unpleasant taste, some patients required placement of an NG tube. NAC rescue was started 6–8 h after AAP treatment.

Two patients experienced grade IV liver toxicity at 20 g/m^2^ AAP and thus 15 g/m^2^ was identified as the maximum tolerated dose; 150 mg/m^2^ was identified as the MTD of BCNU. Mean peak AAP levels in the 15 g/m^2^ AAP cohort was 267 μg/mL. As a correlative biomarker, GSH levels were measured in peripheral blood mononuclear cells (PBMCs) pre- and post-AAP treatment. Interestingly, AAP was shown not to deplete GSH levels in PBMCs—likely because of low CYP2E1 expression in immune cells [19].

Out of the 27 enrolled patients, 2 experienced a partial response (7.4%). The relatively low response rate observed in this phase I trial of AAP could be secondary to the inability to dose-escalate to adequate levels of AAP (1/3 patients in the 20 g/m^2^ cohort experienced a partial response). Thus, with improved rescue regimens, higher AAP doses may be achieved, resulting in improved response rates. Alternatively, AAP may have limited efficacy against melanoma, a tumor that responds to distinct therapies relative to other solid tumors (for instance, melanoma is generally resistant to chemotherapy) [18].

The final clinical case of AAP being used to treat cancer was published in 2005 by Kobrinsky et al. [20]. A 30-month-old child was diagnosed with advanced hepatoblastoma. The patient was initially treated with doxorubicin, but unfortunately progressed. As a second-line therapy, the patient was given 90 mg/m^2^ cisplatin, 1.5 mg/m^2^ vincristine, and 600 mg/m^2^ 5-flurouracil. However, the patient’s tumor proved refractory to this therapy. Finally, the patient was treated with 30 g/m^2^ AAP along with delayed NAC rescue and concurrent cisplatin 90 mg/m^2^. The patient experienced a profound response, with AFP falling from 6700 ng/mL at the start of therapy to 430 ng/mL after the fourth treatment cycle. The patient had his remaining disease surgically resected and only a 5 mm area of viable tumor was present on pathology. At the time of publication, the patient was 8 years out from surgery and disease-free [20] (Table 1).

## 5. Unanswered Questions from Clinical Trials of High-Dose AAP

The clinical trials of high-dose AAP were performed on the basis of theoretical, if unproven, concepts and generally in the absence of pre-clinical data. The goal of the clinical trials was to selectively rescue the toxicity of high-dose AAP using NAC while not reversing AAP’s tumoricidal effects. However, there was no firm pre-clinical basis—beyond theoretical concepts and extrapolations from unrelated data sets—suggesting how this could be accomplished.

Furthermore, the mechanistic rationale cited in the clinical trials for potential AAP efficacy, i.e., that AAP would lead to intra-tumoral GSH depletion and subsequent free radical injury, had not been shown in any pre-clinical models outside of the liver. Indeed, as discussed above, Kobrinsky et al. noted in vitro evidence of anti-leukemic activity of AAP in the absence of GSH depletion [17], and Wolchok et al. found that AAP did not cause GSH depletion in the PBMCs of treated patients, again suggesting an alternative mechanism of the anti-tumor activity of high-dose AAP [18].

In brief, the three key unanswered questions from the clinical trials can be summarized as follows:

1. Does AAP have tumoricidal activity via GSH depletion, i.e., analogous to the mechanism of toxicity in the liver, and if not, what is the mechanism?
2. Is it possible to selectively rescue the normal liver without rescuing the tumoricidal effects of high-dose AAP?
3. What is the optimal rescue regimen that would allow for safe dose escalation of AAP to levels needed for anti-tumor efficacy? Is NAC alone truly the optimal rescue strategy, or are other drugs/combinations of drugs more effective?

As a result of the above unanswered questions, additional clinical trials of high-dose AAP have not been performed despite the promising results of the phase I trials outlined above. Over the last several years, our group has aimed to perform “reverse translational” studies, i.e., perform in vitro and in vivo data laboratory experiments to help explain the promising yet mechanistically nebulous clinical observations.

## 6. High-Dose AAP Selectively Depletes Glutathione in the Liver but Not the Tumor in Pre-Clinical Models

We evaluated the pre-clinical efficacy of high-dose AAP with delayed NAC rescue using hepatocarcinoma and hepatoblastoma models. In these studies, we demonstrated GSH depletion in liver tumor cells in vitro; however, high doses of AAP (5–20 mM) and a relatively long duration of AAP treatment (up to 18 h in cell culture, despite AAP having a half-life of 2–4 h) were used. Our in vitro studies additionally demonstrated synergism between AAP and cisplatin. NAC reversed AAP cytotoxicity towards tumor cells when administered concurrently but demonstrated decreased tumor protection when administered at delayed timepoints in vitro [21].

We subsequently evaluated the efficacy of AAP in a pre-clinical model of ovarian cancer [22,23]. AAP demonstrated increased cytotoxic activity when given with paclitaxel and cisplatin in vitro using the human SKOV3 ovarian cancer cell line. AAP in combination with cisplatin or paclitaxel led to profound loss of mitochondrial membrane potential in treated tumor cells, suggesting that the observed synergy may be mediated by toxicity towards tumor cell mitochondria.

In evaluating the pharmacology of high-dose AAP, we observed that AAP levels were similar in the brain, liver, tumor, and serum of rats treated with high-dose AAP. However, GSH levels were depleted only in the liver but not the tumor, brain, or serum of AAP-treated rats. The finding of selective GSH depletion in the liver but not the tumor was highly novel and directly undermined the proposed mechanism of anti-tumor activity of high-dose AAP, i.e., that it has anti-tumor activity via GSH depletion [23].

**Figure 1.**
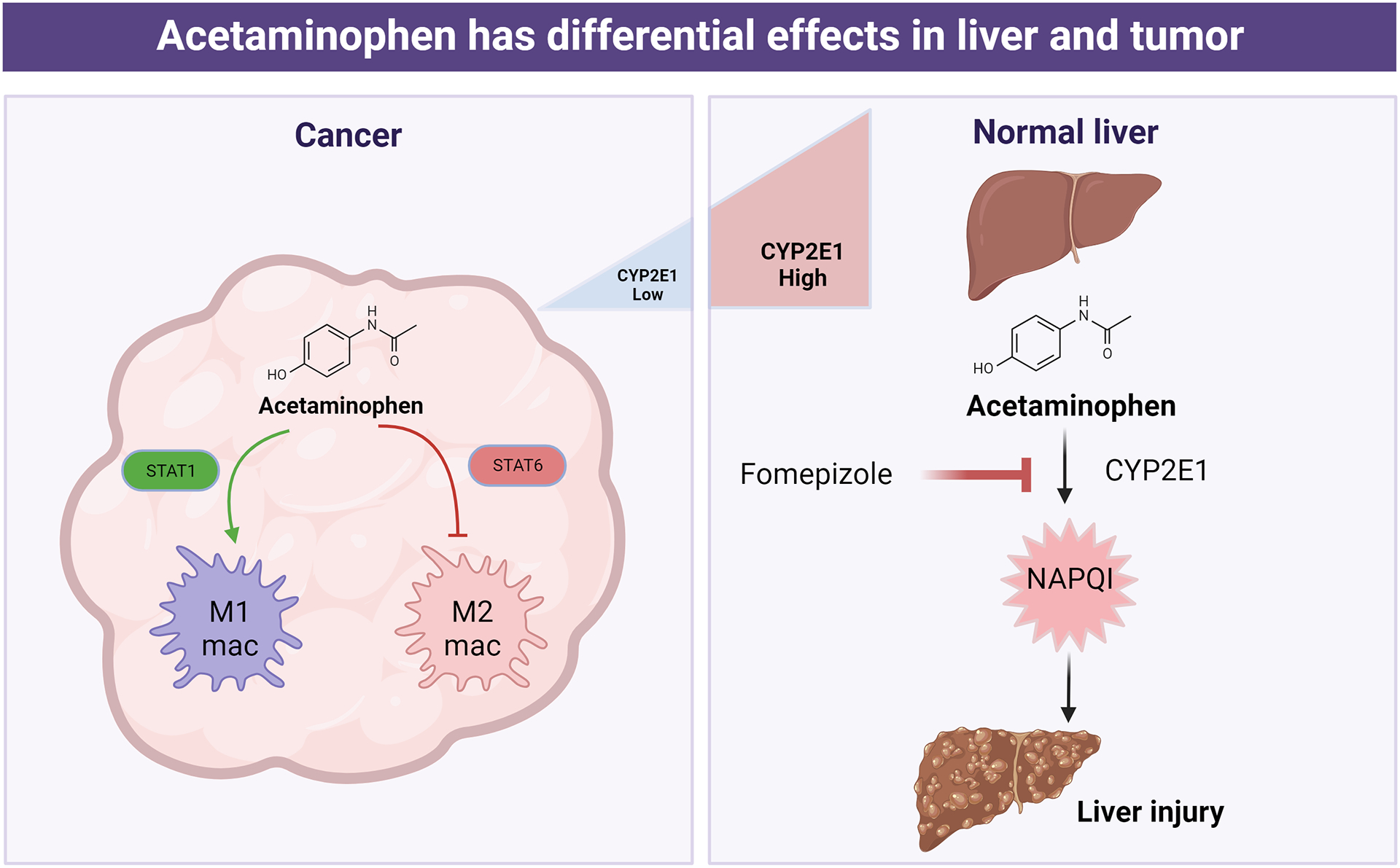
High-dose acetaminophen inhibits M2 polarization of tumor-associated macrophages, leading to anti-tumor immune response. Within the liver, the CYP2E1 inhibitor fomepizole prevents NAPQI formation and resultant toxicity. Differential rescue of liver but not tumor from acetaminophen by fomepizole is a result of selective expression of CYP2E1 in the liver.

Further, our finding that AAP depletes GSH in the liver but not the tumor, subsequently validated in other models [24], suggests a mechanism for selective rescue of normal liver using NAC. NAC functions as a GSH precursor and anti-oxidant; if AAP leads to GSH depletion only in the liver, then NAC can be administered concurrently with high-dose AAP and no tumor protection would be expected. The selective depletion of GSH in the liver upon treatment with high-dose AAP is due to selective expression of CYP2E1 in the liver relative to other organs [19] (Figure 1). If NAC selectively rescues the liver but not the tumor from high-dose AAP treatment, then this raises the possibility of concurrent administration of the two drugs. The potential for AAP administration with concurrent NAC-based rescue could enhance treatment tolerability considering the known improved clinical outcomes when NAC is administered promptly after AAP overdose [3].

## 7. Identification of Free-Radical-Independent Mechanisms of Anti-Tumor Activity of High-Dose AAP

If high-dose AAP does not have anti-cancer activity via GSH depletion and associated free radical injury, then an alternative mechanism must exist. We demonstrated that AAP has anti-cancer stem cell (CSC) activity in multiple pre-clinical models of cancer, an effect that was not reversed by concurrent administration of NAC. Mechanistically, AAP inhibits STAT3 phosphorylation, a protein that is central to the activity and proliferation of CSCs, via direct binding. In STAT3 knockdown lung cancer cells, the anti-CSC effects of AAP are lost, underscoring the physiological relevance of our findings. These data provide additional credence to the concept that NAC may be administered concurrently with AAP for selective rescue from toxicity without compromising anti-tumor activity [24].

JAK-STAT signaling plays a central role in many facets of the anti-tumor immune response. Given our finding that AAP modulates JAK-STAT signaling in tumor cells, we next evaluated the effect of AAP on the adaptive immune system. We observed that AAP inhibits STAT6—involved in pro-tumorigenic M2 polarization of macrophages—but not STAT1—involved in pro-inflammatory anti-tumor M1 macrophage polarization. In vitro and in vivo, high-dose AAP decreased expression of M2 markers in tumor-associated macrophages (such as arginase and CD206) at both the RNA and protein levels. M1 markers, such as iNOS, MHC I, and CD80, were relatively unaffected. Using the mouse syngeneic 4T1 triple negative breast cancer model, it was found that high-dose AAP had profound anti-cancer activity that was lost in macrophage-depleted mice treated with F4/80 antibodies, underscoring the central role of the innate immune system in mediating AAP anti-tumor activity in vivo [25] (Figure 1).

## 8. Optimization of High-Dose AAP Rescue Cocktail

As discussed above, the data supporting NAC as the standard rescue agent for AAP overdose are based largely on single-arm non-randomized trials [8]. Furthermore, there is a paucity of data comparing the efficacy of NAC to alternative rescue agents that have demonstrated pre-clinical promise, such as heparan sulfates [14], 25-hydroxycholesterol-3-sulfate [13], and the CYP2E1 inhibitor fomepizole [26]. Our lab directly compared the most established antidotes to AAP toxicity, NAC and fomepizole, in pre-clinical mouse models. At doses of up to 650 mg/kg, AAP concurrent treatment with fomepizole completely prevented hepatotoxicity. On the other hand, NAC (100 mg/kg IP) provided no significant protection. The lack of observed hepato-protection of NAC in these pre-clinical models is surprising, yet has similarly been observed in other studies [13,27]. Higher doses of NAC (500–1200 mg/kg) have demonstrated improved efficacy in preventing AAP toxicity in mouse models [28,29], but are well beyond the maximal safe dose in humans [30].

**Figure 2.**
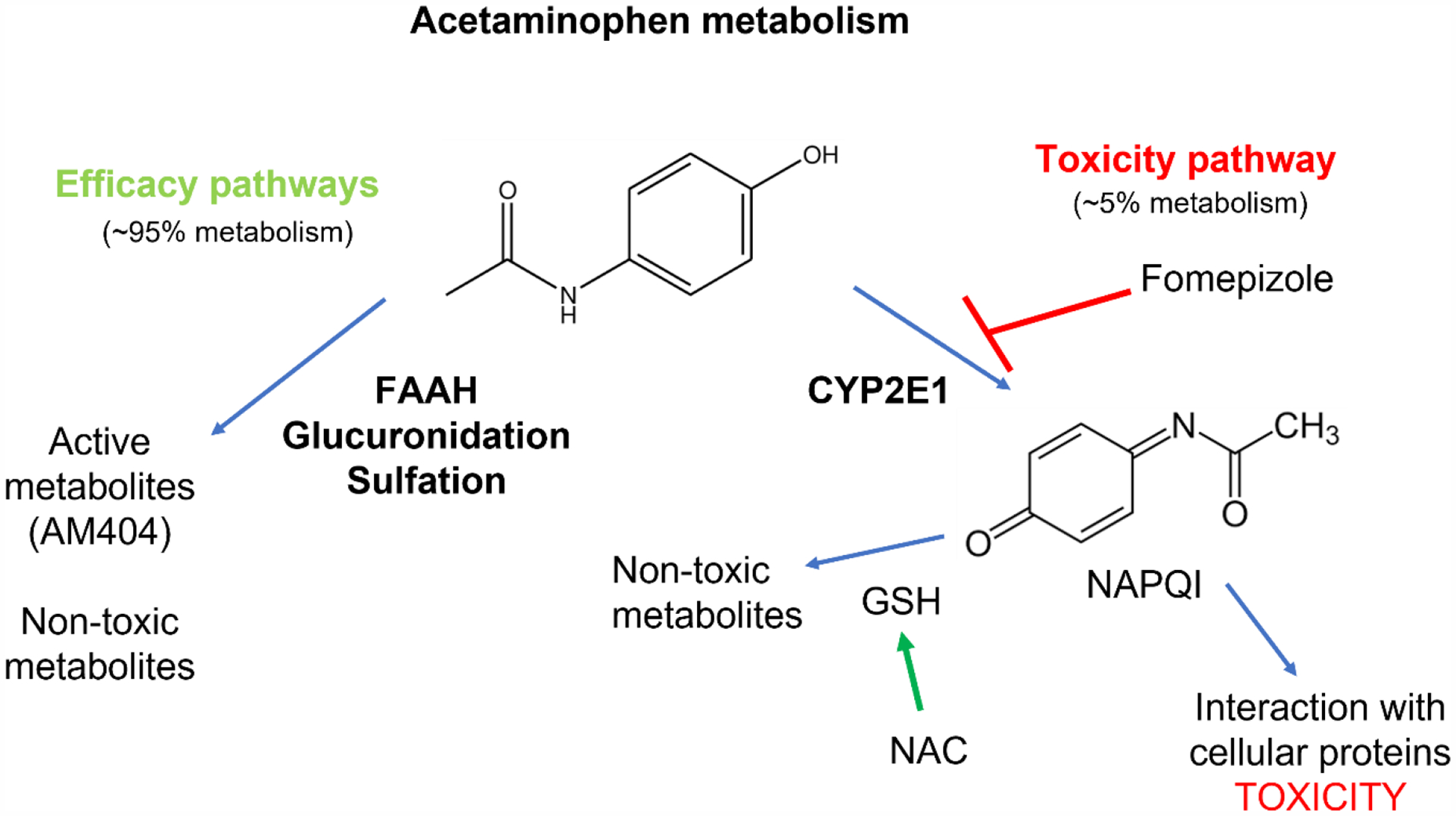
Acetaminophen metabolic pathways. CYP2E1-mediated metabolism is a minor metabolic pathway that leads to toxicity and can be blocked with CYP2E1 inhibitors such as fomepizole to prevent toxicity and preserve anti-tumor efficacy.

CYP2E1 mediates a minor AAP metabolic pathway (about 5–10% of AAP metabolism) that creates a transient reactive intermediate NAPQI that locally binds cellular proteins in the liver, leading to hepatotoxicity. The active AAP metabolite AM404 is generated in a CYP2E1-independent manner (Figure 2) [31]. While fomepizole prevents AAP hepatotoxicity in vivo, fomepizole did not reverse the anti-tumor effects of AAP in vitro or in vivo [32]—likely because CYP2E1 is expressed selectively in the liver (Figure 1) [19]. In fact, using fomepizole-based rescue, we were able to safely dose-escalate AAP to the levels needed for profound anti-tumor activity, 100-fold higher than standard AAP dosing, in commonly used mouse cancer models (LLC lung cancer and 4T1 breast cancer) without any detected toxicity [32].

## 9. Future Directions—AAP as Anti-Cancer Therapeutic

While the precise anti-tumor mechanisms of high-dose AAP are better understood now relative to when the initial clinical trials were conducted in the 1990s [17], much remains to be learned. Animal studies suggest that high-dose AAP has anti-tumor properties through disruption of the JAK-STAT signaling pathway, both in the tumor cell and in tumor-associated macrophages [24,25]. Given the complexity and interconnectedness of the tumor immune microenvironment, it is suspected that other immune cells play important roles in mediating AAP’s anti-tumor immune response. The JAK-STAT signaling pathway, for example, is known to play a role in the maturation of dendritic cells and the activation of helper T cells [33]. Currently, our lab is comprehensively evaluating changes in the tumor immune microenvironment induced by high-dose AAP using such methodologies as single-cell RNA sequencing and multiplex flow cytometry. With improved understanding of the specific molecular interactions at play, patient selection for clinical trials may be optimized, and rationally designed synergistic combinations with other active drugs may be evaluated. Ultimately, improved understanding of high-dose AAP mechanism of action may inform the design of subsequent-generation drugs with improved target specificity and potency.

Additional safety data of high-dose AAP in human patients are needed. In the study by Kobrinsky et al., 19 patients with diverse tumor histologies were treated with high-dose AAP followed by delayed NAC rescue. Dose-limiting liver toxicity was not seen in this study [17]. Subsequently, however, Wolchok et al. observed grade IV liver toxicity in two patients treated with 20 g/m^2^ AAP [18]; the maximum tolerated dose was set at 15 g/m^2^ AAP. In our pre-clinical mouse models, higher doses of AAP could be tolerated without hepatotoxicity when administered with fomepizole-based rescue strategies [32] relative to the NAC regimens used in the initial clinical trials. Human trials are needed to comprehensively evaluate the safety profile, pharmacokinetics, and pharmacodynamics of high-dose AAP with fomepizole-based rescue across a range of doses in patients with advanced cancer.

Early pre-clinical data suggest the efficacy of AAP in diverse cancer models, including hepatic cancer, breast cancer, lung cancer, ovarian cancer, and even atypical teratoid rhabdoid tumor models [21–24]; the use of AAP in brain tumors is particularly compelling, considering that AAP readily crosses the blood–brain barrier. However, the relative efficacy of AAP in various tumor histologies—including solid tumors versus hematologic malignancies—remains to be comprehensively elucidated. Ultimately, large clinical datasets may be needed to assess the clinical efficacy of AAP in diverse malignancies—both alone and in rational combinations with existing anti-tumor agents. Additionally, robust correlative studies from clinical trials will be needed to evaluate the role of JAK-STAT signaling and the immune system in mediating the anti-tumor activity of high-dose AAP.

## 10. Future Directions—Analgesic Potential of AAP/Fomepizole

Patients with advanced cancer often have debilitating pain syndromes associated with their disease, requiring high doses of narcotic pain medicines. Traditional chemotherapy regimens are associated with substantial symptomatic toxicities, including nausea, fatigue, and weakness, that may directly or indirectly exacerbate chronic pain syndromes. The potential of high-dose AAP to not only have anti-cancer activity but also contribute potent analgesic control may be a compelling added benefit of our approach. Kobrinsky et al. noted complete analgesia for 12–24 h in eight patients with chronic pain syndrome receiving high-dose AAP [17] for the treatment of advanced cancer. As described above (Figure 2), AM404 is an active analgesic metabolite of AAP that is created in a CYP2E1-independent fashion via deacetylation of AAP to para-aminophenol followed by fatty acid amide hydrolase (FAAH)-catalyzed conjugation with arachidonic acid [34]. Thus, the CYP2E1 inhibitor fomepizole is unlikely to mitigate the analgesic effects of AAP, and high-dose AAP with fomepizole rescue may be a potent analgesic cocktail that spares the toxicities of narcotics, such as constipation, respiratory depression and ultimately dependance. Further research studying the analgesic benefits of high-dose AAP with fomepizole-based rescue will require detailed evaluation in both animal and human studies.

## 11. Conclusions

High-dose AAP has demonstrated promise in phase I clinical trials for the treatment of patients with advanced malignancy. Lack of understanding of therapeutic mechanisms has limited the subsequent development of AAP as an anti-cancer drug. More recently, “reverse translational” pre-clinical studies have provided substantial mechanistic insights that may facilitate future clinical trials of high-dose AAP in patients with cancer. Most importantly, recently published data provide evidence that AAP may be administered concurrently with highly effective antidotes such as fomepizole to prevent toxicity without compromising therapeutic anti-tumor efficacy. With these recent advances, we believe it is imperative that high-dose AAP with fomepizole-based rescue be evaluated in clinical trials for patients with advanced cancer.

## Figures and Tables

**Table 1.** Clinical experience of high-dose acetaminophen in cancer patients.

Citation	Intervention	Duration	Patients Enrolled (*n*)	Outcomes
Kobrinsky, 1996 [17]	Oral AAP with dose escalation and delayed NAC rescue for diverse malignancies.	Until disease progression or unacceptable toxicity	19	Toxicity: No dose-limiting toxicities at doses of up to 20 g/m^2^ AAP. Efficacy: Three out of fourteen evaluated patients (21%) had a partial response.
Wolchok, 2003 [18]	Carmustine dose escalation, oral AAP dose escalation, and delayed NAC rescue for advanced malignant melanoma.	Until disease progression or unacceptable toxicity	27	Toxicity: Two patients experienced grade IV liver toxicity at 20 g/m^2^ AAP, so 15 g/m^2^ was the maximum tolerated dose (in combination with carmustine). Efficacy: Two patients had a partial response.
Kobrinksy, 2005 [20]	Case report of treatment of progressive hepatoblastoma with high-dose (30 g/m^2^) AAP, NAC, and cisplatin.	Four cycles, then surgical resection of tumor	1	Toxicity: No toxicities noted. Efficacy: Near-complete response followed by resection of residual necrotic tumor. Patient disease-free for 8 years at time of publication.

## Data Availability

Data is contained within the article.
